# Genetic structure of brown pelicans (*Pelecanus occidentalis*) in the northern Gulf of Mexico in the context of human management and disturbance

**DOI:** 10.1371/journal.pone.0185309

**Published:** 2017-10-04

**Authors:** Brock Geary, Susan M. Longest, Kym Ottewell, Samantha M. Lantz, Scott T. Walter, Jordan Karubian, Paul L. Leberg

**Affiliations:** 1 Department of Ecology & Evolutionary Biology, Tulane University, New Orleans, Louisiana, United States of America; 2 Department of Biology, University of Louisiana at Lafayette, Lafayette, Louisiana, United States of America; Department of Agriculture and Water Resources, AUSTRALIA

## Abstract

Environmental disturbances, both natural and anthropogenic, have the capacity to substantially impact animal behavior and abundance, which can in turn influence patterns of genetic diversity and gene flow. However, little empirical information is available on the nature and degree of such changes due to the relative rarity of longitudinal genetic sampling of wild populations at appropriate intervals. Addressing this knowledge gap is therefore of interest to evolutionary biologists, policy makers, and managers. In the past half century, populations of the brown pelican (*Pelecanus occidentalis*) in the southeastern United States have been exposed to regional extirpations, translocations, colony losses, and oil spills, but potential impacts on genetic diversity and population structure remain unknown. To investigate the cumulative impacts of recent disturbances and management actions, we analyzed seven microsatellite loci using genetic samples collected from 540 nestlings across twelve pelican colonies from two time periods, corresponding to before (n = 305) and after (n = 235) the 2010 Deepwater Horizon oil spill. Pre-2010 populations in Texas were significantly differentiated from Louisiana, Alabama, and Florida populations to the east, with reintroduced populations in southeastern Louisiana having less genetic diversity than sites in Texas, consistent with a recent bottleneck. In contrast, there was no evidence of a geographic component to genetic structure among colonies sampled after the spill, consistent with increased dispersal among sites following the event. This pattern may be associated with reduced philopatry in response to colony abandonment in the areas most heavily impacted by the Deepwater Horizon event, though other factors (e.g., rehabilitation and translocation of oiled birds or colony loss due to erosion and tropical storms) were likely also involved. Future monitoring is necessary to determine if bottlenecks and loss of genetic variation are associated with the oil spill over time, and is recommended for other systems in which disturbance effects may be inferred via repeated genetic sampling.

## Introduction

Regional patterns of intraspecific genetic diversity are of great interest to both evolutionary and conservation biologists, as populations lacking sufficient genetic variability are susceptible to an accumulation of fixed deleterious alleles via inbreeding and/or genetic drift, which may result in decreased adaptability, fitness, and probability of persistence [1–3]. Information on regional population genetic structure can also contribute to our understanding of how biogeographic, behavioral, and anthropogenic effects may shape evolutionary processes in populations of interest, including those of conservation concern [4,5]. Obtaining this information is therefore a frequent priority for researchers seeking to characterize a population’s status and understand conservation issues acting at the local or regional scale.

While genetic methods are often employed in ecological studies, variation in genetic parameters over ecologically relevant time scales has received relatively little attention compared to 'snapshot' studies, in which a single time frame is characterized with little information on how genetic patterns may be changing over time [6,7]. Among those studies that have taken a multi-year approach, many have detected decreases in genetic diversity over time across a variety of taxa, including many threatened and endangered species [8–13]. Occasionally, however, no changes are recorded between samples over time [12,14,15]. Expanded information on genetic change over time is thought to be important in the context of persistent anthropogenic disturbance and resource exploitation across the world’s ecosystems [7,16–18], but investigations of changes over shorter time scales remain rare [18]. In a management framework, these changes can be used to assess population status, as altered rates of gene flow across a region can impact population structure and subsequent local adaptation across a region, or demonstrate the effects of environmental changes, including habitat alteration [19–21].

Population genetic data can also be leveraged to investigate the impacts of large regional disturbances and human responses on genetic diversity and structure [6,22–24], but such studies are relatively rare. For example, following the 2010 Deepwater Horizon oil spill off the coast of Louisiana, USA, immediate effects (e.g. survival, reproduction, and/or behavior of affected organisms) were well-quantified in both field and experimental settings [25–36], but there are few published investigations related to pre- and post-spill differences in animal diversity and/or genetic structure (but see [37]). For these reasons, longitudinal genetic monitoring over shorter time scales (e.g. years or generations rather than decades) can be regarded as a useful, but infrequently utilized tool by which to evaluate the effects of a disturbance or management project [13,38]. In the case of reintroduction efforts of extirpated species, using adequate numbers of translocated individuals and source populations is crucial to the avoidance of harmful founder effects and subsequent biological processes such as inbreeding depression [39].

The eastern brown pelican (*Pelecanus occidentalis carolinensis*) is a widespread and iconic seabird whose northern Gulf of Mexico populations declined precipitously in the mid-1900s in response to anthropogenic stressors, leading to extirpation in the state of Louisiana by 1963 [40]. Reintroductions in Louisiana began in 1968 [41,42], with 1,200 pelicans brought from six Florida source populations to Louisiana over a thirteen year period [43,44]. The species has since established new nesting colonies in Louisiana [44], and its global population has grown to levels sufficient for its removal from the Endangered Species list in 2009 [45], but the degree to which Louisiana populations may have experienced genetic bottlenecks, particularly those associated with reintroduction, remains unclear. Additionally, the Deepwater Horizon oil spill in the year following delisting had immediate negative impacts on seabird and wading bird populations in the northern Gulf of Mexico, including brown pelicans [29,30]. These impacts include acute and direct mortality, as well as sub-lethal effects on behavior and condition, but as with most taxa the magnitude and nature of spill-related impacts for brown pelicans remain unclear. For example, oiling of important pelican breeding colonies may have impacted site fidelity and, consequently, gene flow and population structure across the northern Gulf. Distinctive patterns of differentiation between colonies among pre- vs. post-2010 sampling efforts would be consistent with localized effects on distinct subpopulations eliminating unique genotypes via colony loss or increased abandonment rates. Additionally, genetic analyses to detect possible founder effects would be useful to accurately gauge the success of historical pelican management efforts. However, a thorough genetic assessment of brown pelicans in the southeastern United States, including tests for effects brought about by reintroductions in Louisiana and subsequent regional disturbances, is currently lacking. This represents an important knowledge gap, as the order Pelecaniformes has previously been identified as a group in need of broader population genetic assessment among seabirds [46], and the continuation of monitoring efforts should be a priority for determining the effects of the Deepwater Horizon event and other anthropogenic impacts on these and other Gulf populations.

Our objectives in this study were to quantify genetic diversity and population structure across the range of the brown pelican in the southeastern United States, and to assess potential founder effects and subsequent bottlenecks that may be associated with reintroductions to Louisiana from Florida. We also characterize changes in population structure and diversity between two time periods that bracket the Deepwater Horizon oil spill to better understand if demographic changes resulting from this event resulted in a genetic signature.

## Materials and methods

### Sampling and microsatellite development

**Fig 1:**
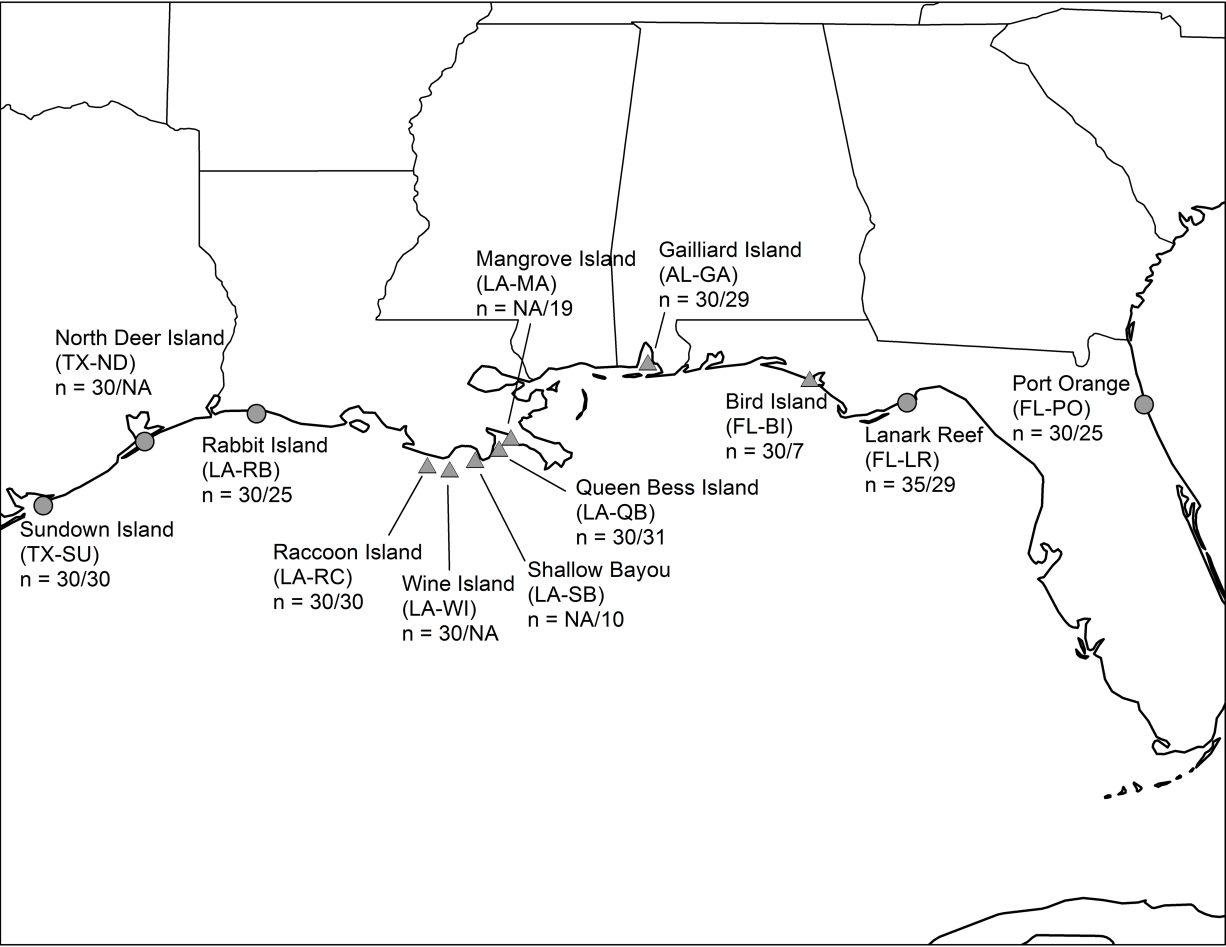
Brown pelican sampling locations in the southeastern United States, with colony abbreviations. Backslash separates pre- and post-2010 sample sizes (NA = not sampled in that time period). Triangles indicate colonies that were oiled during the Deepwater Horizon spill.

Initial sampling for a study of pelican genetic structure occurred throughout the northern Gulf of Mexico region from 2007–2010 (n = 305 samples). We hereafter refer to these samples as “pre-2010” as we sampled all 2010 individuals well before Deepwater Horizon oil slicks in 2010 reached the colonies under examination. We sampled the region again in 2011 (n = 235, hereafter “post-2010”), including 8 of the 10 colonies sampled pre-2010. We sampled chicks aged 3–9 weeks (all flightless [40]), in June and July of each year, from a total of 12 breeding colonies across the Gulf of Mexico and the Atlantic coast of Florida (Fig 1). We sampled one chick per nest to avoid excess disturbance and to avoid inclusion of siblings in our samples, and released all chicks immediately following sampling.

We collected blood samples from the brachial vein in 75 mm capillary tubes and stored them in 600μL cell lysis solution until DNA could be extracted. We performed DNA extractions using Qiagen DNeasy Blood and Tissue Kits following manufacturer protocols, and isolated novel microsatellite loci for the species from a single individual using 454 sequencing [47] at the Sequencing and Genotyping Core at the University of California Los Angeles (see S1 File for additional information). We ultimately found five polymorphic loci for genotyping, and added two loci from other pelican species: one from American white pelicans (*Pelecanus erythrorhynchos*) [48], PeEr04, and one from great white pelicans (*Pelecanus onocrotalus*), Pel086 [49]. Probabilities of identity for all populations were < 0.002, suggesting that these loci are sufficient to make population inferences. We assessed scoring errors, allelic dropout, and potential null alleles using MICROCHECKER ver. 2.2.3 [50]. We used GenAlEx version 6.501 [51,52] to check for deviations from Hardy-Weinberg equilibrium. We observed significant deviations after sequential Bonferroni correction in 5 of 69 pre-2010 locus-colony populations and 4 of 68 post-2010 polymorphic locus/population combinations. We retained all loci for analysis, as no consistent patterns of departure occurred for any loci across multiple populations.

### Ethics statement

This study was carried out in accordance with field and laboratory protocols approved by the Institutional Animal Care and Use Committees of Tulane University (protocol no. 0395) and the University of Louisiana at Lafayette (protocol nos. 2009-8717- 075, 2010-8717-068, and 2011-8717-065). Sampling efforts on individual colonies were limited by land manager recommendations, and all efforts were made during field sampling to minimize stress to study individuals and the colonies at large. Additionally, island access and sampling on protected or managed islands were approved by the Texas Parks and Wildlife Department (permit no. SPR-0410-046), Louisiana Department of Wildlife and Fisheries (permit nos. LNGP-08-009, LNGP-08-010, LNGP-09-52, LNHP-10-033, and LNHP-11-31), Alabama Department of Conservation and Natural Resources (Wildlife and Freshwater Fisheries Division, permit no. MB182448-0), and Florida Fish and Wildlife Conservation Commission (permit nos. LSSC-11-00074, LSSC-11-00075).

### Assessment of genetic diversity within populations

We used GenAlEx to calculate several basic genetic parameters in both samples: number of alleles, observed and expected heterozygosity (H_O_ and H_E_, respectively), and inbreeding coefficients (F_IS_). We calculated allelic richness (A_R_) for each population and locus using the program ADZE version 1.0 [53]. We used randomized block ANOVA, blocking by locus, to test the null hypothesis that estimates of A_R_ and H_E_ did not differ among population samples for either of the time periods. In analyses where we identified differences in genetic diversity estimates among samples, we used a Tukey test to determine statistical significance. For those breeding colonies where we had >25 samples in a time period, we assessed signatures of the species’ reintroduction using BOTTLENECK version 1.2.02 [54]. We used a two-phase model of evolution, considered to model microsatellite evolution more realistically than stepwise or infinite allele models [54]. We performed 10,000 simulations for each sample (9,000 single-step mutations, 1,000 multistep mutations), and 10% variance among multiple steps to conduct a one-tailed Wilcoxon sign-rank test for excess heterozygosity (indicative of a bottleneck) for each population. We also examined allele frequency distributions to determine whether they were approximately L-shaped, indicating mutation-drift equilibrium. Deviation from this distribution would indicate a bottleneck via a mode shift in the distribution [55].

### Assessment of genetic structure among populations

To investigate regional population structure, we applied several analyses to avoid potential issues of reliability associated with over-dependence on individual metrics or software [56]. We calculated pairwise genetic differentiation by calculating theta (hereafter F_ST_) [57] in GENEPOP version 4.2.1 [58] with the Fisher approach, assessing significance of pairwise differentiation with sequential Bonferroni adjustment for multiple comparisons [59]. To assess potential isolation by distance, as well as the relationship between pairwise colony differentiation over time for the colonies sampled in both periods, we performed Mantel tests [60] on pairwise differentiation (as F_ST_/(1-Fst)) matrices and linear geographic distances between each colony pair [61]. We ran Mantel tests with 999 permutations in GenAlEx to determine the significance of the association between the two matrices. We used the Bayesian clustering programs STRUCTURE version 2.3.4 [62] and TESS version 2.3.1 [63,64] to infer population structure in both time periods [65]. We ran STRUCTURE using the admixture and correlated allele frequencies models, with a burn-in of 500,000 and data collection of 500,000 replicates, using 10 iterations each of K values (assumed numbers of genetically distinct clusters) of 1–10. We conducted this analysis both with and without using sampling location to aid in the clustering of samples; because all sampled individuals were born at the sampling sites, the use of sampling location to aid in the clustering may be more appropriate than if the samples had consisted of post-fledging birds. We used STRUCTURE Harvester [66] to obtain an estimate of the number of clusters using the Evanno method [67] along with the likelihood estimates of each K value [62]. We also used the CLUMPAK service [68] to implement the programs CLUMPP [69] and DISTRUCT [70] to display probabilities of cluster membership for each individual based on STRUCTURE results. We ran TESS using the BYM [71] and admixture models, with a burn-in of 10,000 and 50,000 total sweeps per run, using K values of 2–10 with 100 iterations each. We then used the iterations with the lowest 20% of DIC values for suggested values of K to visualize results of interest, again using CLUMPP and DISTRUCT.

We used non-metric multi-dimensional scaling (NMDS) to visualize relationships among populations in both the pre- and post-2010 samples [72]. We generated a F_ST_ matrix for each sampling period in GENEPOP, and imported matrices to program R version 3.4.0 [73] for ordination. We performed a two-dimensional visualization of these data using the isoMDR function in the package ‘MASS’ [74].

### Analysis of differences between time periods

We quantified changes in A_R_ and H_E_ between the time periods using ANOVA, blocked by locus. To test for temporal changes in allele frequencies from each sampling location, we used Fisher’s test, implemented in GENEPOP. To assess temporal change in allele frequencies across loci, we estimated F_C_, an index of temporal allele frequency change [75,76], for populations where we had samples in both time periods. To avoid biases associated with rare alleles [77], we also recalculated F_C_ while excluding alleles with frequencies of < 0.05 in any population; however, this exclusion had no effect on the results of comparisons among populations. To ensure that changes between pre- and post-2010 periods were not affected by a pre-2010 colony’s sampling year, we conducted a simple linear regression to correlate Fc with the interval between sampling bouts for each colony; this relationship was non-significant (R^2^ = 0.290, P = 0.169).

## Results

### Pre-2010 samples

#### Within-population diversity

There were statistically significant differences among populations in the pre-2010 population samples for estimates of A_R_ and H_E_ (F_9,54_ = 3.343, P = 0.003, and F_9,54_ = 4.940, P < 0.001, respectively). The general pattern was for less genetic diversity in some sites in southeastern Louisiana relative to samples from Texas (Table 1). The two samples from Texas also had significantly higher estimates of H_E_ than the samples from Galliard, Queen Bess, and Wine Islands (Table 1), all of which are reestablished populations. There was no evidence for large deviations in F_IS_ from zero in any of the populations (Table 1). BOTTLENECK analyses found no evidence of significant excess of heterozygosity, and therefore no signal of a founder effect, in any of the sampled populations.

**Table 1:** Population genetic diversity statistics for brown pelican chicks sampled in the northern Gulf of Mexico.

Population	Sampling period	n	N_A_	A_R_	H_O_	H_E_	F_IS_
***West of Oiled Areas***							
TX-SU	Pre-2010	30	4.43 (0.48)	2.58 (0.32)_A_	0.49 (0.10)	0.48 (0.08)_A_	-0.002 (0.055)
	Post-2010	30	4.00 (0.66)	2.55 (0.32)	0.42 (0.07)	0.48 (0.08)	0.084 (0.094)
TX-ND	Pre-2010	30	4.14 (0.51)	2.56 (0.36)_A_	0.49 (0.08)	0.47 (0.09)_AB_	-0.078 (0.046)
LA-RB	Pre-2010	30	3.86 (0.74)	2.45 (0.45)_AB_	0.37 (0.09)	0.41 (0.11)_AB_	0.074 (0.046)
	Post-2010	25	3.14 (0.63)	2.26 (0.43)	0.41 (0.13)	0.38 (0.11)	-0.041 (0.103)
***Oiled Areas***							
LA-RC	Pre-2010	30	3.86 (0.60)	2.36 (0.38)_AB_	0.42 (0.10)	0.42 (0.10)_AB_	-0.012 (0.032)
	Post-2010	30	3.71 (0.94)	2.20 (0.40)	0.33 (0.10)	0.36 (0.10)	0.040 (0.077)
LA-WI	Pre-2010	30	3.14 (0.74)	2.11 (0.44)_B_	0.38 (0.13)	0.34 (0.12)_C_	-0.097(0.026)
LA-SB	Post-2010	10	3.29 (0.61)	2.44 (0.45)	0.37 (0.10)	0.39 (0.10)	-0.003 (0.079)
LA-QB	Pre-2010	30	3.43 (0.72)	2.12 (0.46)_B_	0.31 (0.11)	0.32 (0.12)_C_	-0.014 (0.042)
	Post-2010	31	3.29 (0.75)	2.12 (0.43)	0.32 (0.11)	0.34 (0.11)	0.005 (0.065)
LA-MA	Post-2010	19	3.57 (0.84)	2.28 (0.43)	0.37 (0.10)	0.38 (0.11)	0.047 (0.111)
AL-GA	Pre-2010	30	3.29 (0.68)	2.19 (0.45)_AB_	0.36 (0.12)	0.36 (0.12)_C_	-0.016 (0.047)
	Post-2010	29	3.43 (0.48)	2.37 (0.38)	0.38 (0.08)	0.41 (0.10)	0.046 (0.080)
FL-BI	Pre-2010	30	3.58 (0.78)	2.28 (0.47)_AB_	0.37(0.12)	0.37 (0.12)_AB_	-0.008 (0.023)
	Post-2010	7	2.71 (0.47)	2.30 (0.35)	0.42 (0.09)	0.39 (0.09)	-0.120 (0.080)
***East of Oiled Areas***							
FL-LR	Pre-2010	35	4.00 (0.82)	2.34 (0.48)_AB_	0.33 (0.11)	0.38 (0.12)_AB_	0.085 (0.065)
	Post-2010	29	3.57 (0.65)	2.17 (0.41)	0.37 (0.12)	0.36 (0.11)	-0.047 (0.056)
FL-PO	Pre-2010	30	4.00 (0.82)	2.34 (0.45)_AB_	0.37 (0.10)	0.39 (0.11)_AB_	0.029 (0.024)
	Post-2010	25	3.14 (0.67)	2.32 (0.44)	0.36 (0.11)	0.41 (0.11)	0.100 (0.122)
**MEAN (SE)**	**Pre-2010**	**305**	**3.77 (0.21)**	**2.35 (0.06)**	**0.39 (0.03)**	**0.39 (0.03)**	**-0.004 (0.014)**
	**Post-2010**	**235**	**3.61 (0.15)**	**2.30 (0.04)**	**0.38 (0.02)**	**0.39 (0.02)**	**0.006 (0.015)**

Diversity values (± SE): n = number of individuals sampled, N_A_ = number of alleles, A_R_ = allelic richness, H_O_ = observed heterozygosity, H_E_ = expected heterozygosity, F_IS_ = inbreeding coefficient. Subscripts in pre-2010 populations associated with estimates of A_R_ and H_E_ reflect results of a Tukey multiple comparison test; values with the same letter are not statistically differentiated (alpha = 0.05). No post-2010 samples were differentiated using the same test.

#### Population structure

**Fig 2:**
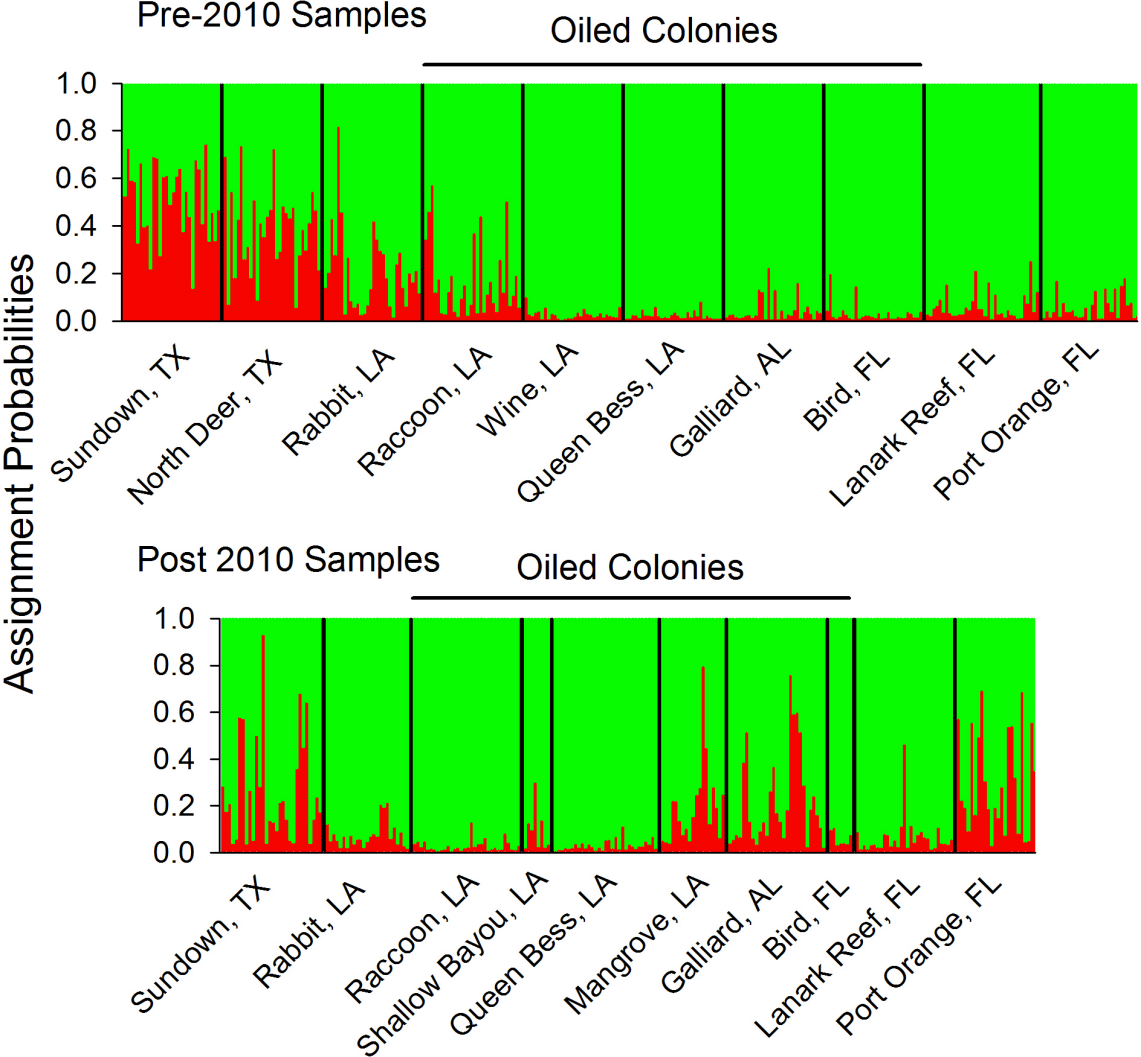
TESS assignments of individual pelicans from pre-2010 and post-2010 samples. Colony abbreviations can be found in Fig 1.

**Fig 3:**
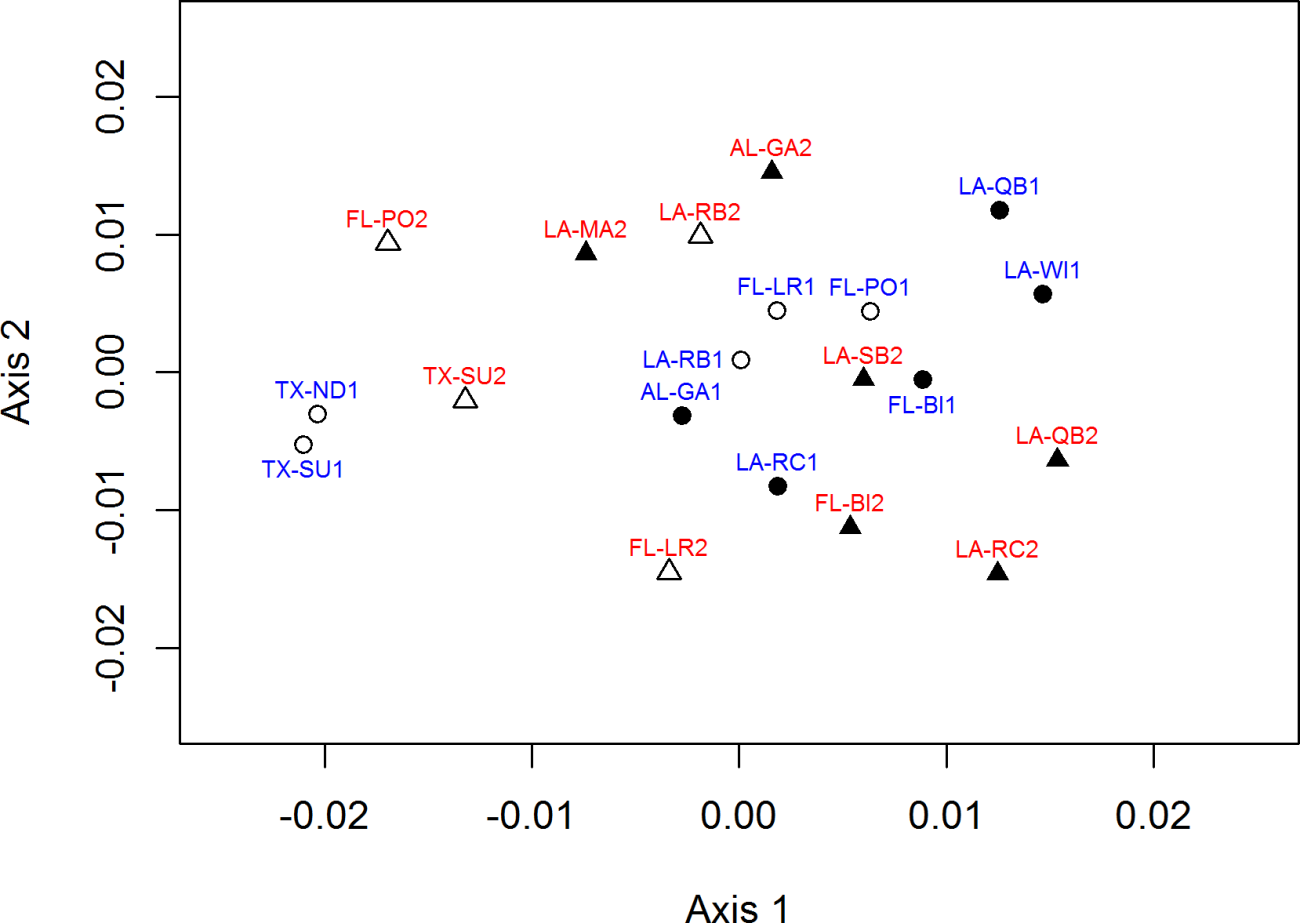
Nonmetric multidimensional scaling ordination of pre- and post-2010 populations of pelicans based on genetic divergence. Pre-2010 colonies are represented with a “1” suffix, blue text, and circles. Post-2010 colonies are represented with a “2” suffix, red text, and triangles. Filled symbols represent colonies oiled during the Deepwater Horizon spill.

Pairwise pre-2010 population differentiation was relatively low, but statistically significant among several population pairs (Table 2). All of these differences occurred between the Texas populations and those from southeastern Louisiana to western Florida. Although there was a geographic component to the differentiation, there was no significant association between pairwise F_ST_ and geographic distance values (R^2^ = 0.030, P = 0.173). With STRUCTURE analysis accounting for information on sampling location, the Evanno approach suggested the optimal number of clusters (K) was two. However, examining changes in the likelihood of different numbers of K suggested that there was no strong support for more than one cluster, a result supported by examination of individual assignments, which also did not provide evidence for multiple clusters. When we did not use sampling location as part of the analysis, both the Evanno approach and examination of likelihoods suggested there were two clusters present in the data, but examination of individual assignments still did not provide clear patterns of structuring in the data. In contrast, TESS results indicated that as many as six clusters were present. Individual TESS assignment plots from DISTRUCT with K = 2, the number suggested by STRUCTURE, showed partial separation between the two Texas colonies and the others to the east, with admixture in the western Louisiana colonies (Fig 2). Plotting with K = 6 did not change the overall pattern of assignments, with two clusters still predominating (See S2 File). NMDS ordination of the colonies supported this structure, with the two Texas colonies clearly separating from the others (TX-ND1 and TX-SU1; Fig 3).

**Table 2:** Pre-2010 pairwise estimates of differentiation.

	TX-SU	TX-ND	LA-RB	LA-RC	LA-WI	LA-QB	AL-GA	FL-BI	FL-LR	FL-PO
**TX-SU**	—	0.9873	0.0499	0.0412	**<0.0001**	**<0.0001**	**0.0011**	**0.0003**	0.0041	0.0107
**TX-ND**	0.000	—	0.0067	0.1342	**0.0005**	**<0.0001**	0.0014	0.0023	0.1159	0.0663
**LA-RB**	0.008	0.007	—	0.2189	0.1910	0.0500	0.1250	0.1032	0.0802	0.2386
**LA-RC**	0.008	0.010	0.000	—	0.0991	0.0185	0.1474	0.0340	0.0808	0.3636
**LA-WI**	**0.043**	**0.037**	0.001	0.016	—	0.7704	0.4725	0.7869	0.3866	0.8622
**LA-QB**	**0.047**	**0.043**	0.004	0.019	0.005	—	0.0794	0.4851	0.2925	0.3344
**AL-GA**	**0.017**	0.016	0.000	0.003	0.008	0.013	—	0.0895	0.3375	0.2077
**FL-BI**	**0.027**	0.025	0.000	0.005	0.000	0.003	0.000	—	0.4948	0.9175
**FL-LR**	0.019	0.013	0.000	0.005	0.000	0.000	0.000	0.000	—	0.7338
**FL-PO**	0.020	0.018	0.000	0.000	0.000	0.000	0.002	0.000	0.000	—

F_ST_ (below the diagonal) and p-values of pair-wise Fisher tests of population differentiation (above the diagonal) for brown pelican populations in the northern Gulf of Mexico sampled before and after 2010. P-values in bold were significant following sequential Bonferroni adjustment.

### Post-2010 samples

#### Within-population diversity

Unlike the pre-2010 samples, there were no differences in A_R_ or H_E_ among populations after 2010 (F_9,54_ = 1.346, P = 0.236, and F_9,54_ = 1.347, P = 0.236, respectively; Table 1). As in the case of the pre-2010 samples, there was no evidence that any of the populations experienced excess heterozygosity or allelic mode shifts associated with a recent population bottleneck.

#### Population structure

The sizes of differences in gene frequencies, as measured by F_ST_, among post-2010 samples were similar to those among pre-2010 samples (Table 3). However, the pattern of those differences was not consistent. Unlike the pre-2010 samples, many of the significant differences in allele frequencies were observed among sites within southeastern Louisiana and between those sites and Port Orange on the Atlantic coast of Florida. As in the case of the pre-2010 samples, there was no association between pairwise estimates of F_ST_ and geographic distance (R^2^ = 0.025, P = 0.174).

**Table 3:** Post-2010 pairwise estimates of differentiation.

	TX-SU	LA-RB	LA-RC	LA-SB	LA-QB	LA-MA	AL-GA	FL-BI2	FL-LR2	FL-PO2
**TX-SU**	—	0.0061	**<0.0001**	0.3858	**0.0001**	0.1461	0.0408	0.3514	0.0078	0.1478
**LA-RB**	0.012	—	0.0231	0.4475	0.0788	0.0686	0.0026	0.2288	0.0148	**0.0005**
**LA-RC**	**0.022**	0.026	—	0.9072	0.8542	**0.0008**	**0.0009**	0.1680	0.0312	**<0.0001**
**LA-SB**	0.007	0.008	0.000	—	0.6293	0.3494	0.4611	0.7634	0.4558	0.0741
**LA-QB**	**0.028**	0.019	0.000	-0.003	—	0.0024	0.0028	0.3339	0.0499	**<0.0001**
**LA-MA**	0.000	0.000	**0.029**	0.008	0.026	—	0.2710	0.1361	0.2373	0.5424
**AL-GA**	0.007	0.010	**0.022**	0.000	0.018	-0.001	—	0.2312	0.0055	0.1096
**FL-BI**	0.000	0.022	0.010	0.000	0.000	0.015	0.002	—	0.5931	0.0333
**FL-LR**	0.013	0.023	0.010	0.008	0.016	0.007	0.023	0.006	—	0.0314
**FL-PO**	0.000	**0.020**	**0.043**	0.015	**0.045**	0.000	0.009	0.029	0.012	—

F_ST_ (below the diagonal) and p-values of pair-wise Fisher tests of population differentiation (above the diagonal) for brown pelican populations in the northern Gulf of Mexico sampled before and after 2010. P-values in bold were significant following sequential Bonferroni adjustment.

The STRUCTURE analysis of the post-2010 samples was similar to the analysis for the pre-2010 samples. The Evanno approach suggested K = 3 with sampling location informing the analysis and K = 2 without; however, likelihood estimates and individual assignments suggested there was only one population cluster. In contrast to the pre-2010 analyses, examination of assignment plots in TESS based on runs with the lowest DIC values also did not yield a clear suggestion of any structuring. The NMDS ordination (Fig 3; red labels) also did not show any clear separation or clustering among any populations in relation to their geographic locations.

### Comparison of pre-2010 and post-2010 samples

**Fig 4:**
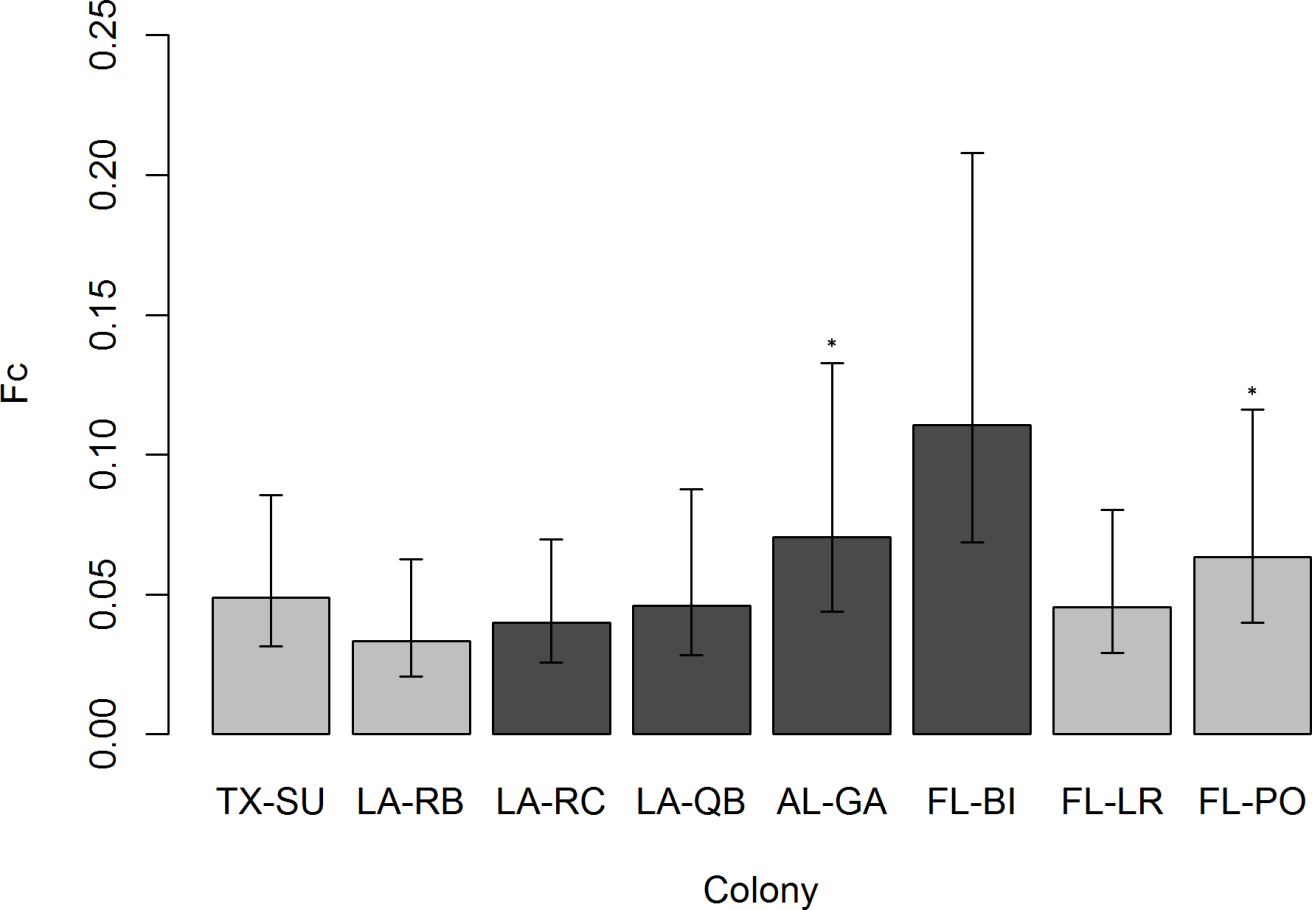
F_C_ values for the eight colonies in which sampling occurred during both periods. Error bars represent 95% confidence intervals. Colonies oiled during the Deepwater Horizon spill are in dark grey, and those with significantly different allele frequencies following sequential Bonferroni adjustment are denoted with an *.

There was no evidence of loss of genetic diversity between the pre-2010 and post-2010 samples among the seven populations sampled in both time periods (A_R_: F_1,90_ = 0.628, P = 0.430; H_E_: F_1,90_ = 0.002, P = 0.965) and no significant interactions between time period and population (A_R_: F_7,90_ = 0.616, P = 0.741; H_E_: F_7,90_ = 0.629, P = 0.731). There were considerable changes in allele frequencies in all the populations, but the magnitude of the change was much larger in some populations than others. Allele frequencies were significantly different, following sequential Bonferroni adjustment of error rates, for Galliard Island in Alabama (P < 0.001) and Port Orange Island in Florida (P = 0.004). The estimates of F_C_ reflected this pattern, with the largest values of change seen in Galliard, Port Orange, and Bird Islands (Fig 4), the latter of which was not significant due to the small size of the post-2010 sample.

## Discussion

By comparing multiple populations of a colonial seabird at two time points, we provide insights into the degree to which gene frequencies and diversity have shifted in the Gulf of Mexico region over time scales of ecological interest. Our data indicate a minor, if any, lasting genetic legacy from a species reintroduction that occurred 10–15 generations prior to our study, but are consistent with notable shifts in gene frequencies between two sampling points that bracket a major environmental disturbance, the Deepwater Horizon oil spill. This study underscores the importance of longitudinal comparisons when assessing regional genetic diversity and structure of wild populations, especially when large-scale disturbances have the potential to alter these patterns.

The brown pelican is a wide-ranging seabird with occasional reported movements on the scale of thousands of kilometers [78,79], so we expected to find high genetic diversity and low population structure across the northern Gulf of Mexico region. However, based on reports of high natal philopatry [25,40], we predicted that we would detect the effects of founder events associated with the reestablishment of the Louisiana populations 40–50 years (i.e., 10–15 generations) prior to sample collection, including a signature of a bottleneck and increased similarity among restored Louisiana and source Florida populations relative to Texas populations.

Overall, despite relatively high levels of genetic diversity among the contemporary brown pelican population in the northern Gulf compared to other seabird studies [80,81], there were some notable spatial differences among the pre-2010 populations. The pre-2010 populations on Wine and Queen Bess Islands in southeastern Louisiana exhibited the two lowest observed levels of allelic richness, and were among the least heterozygous of all sites, but were only significantly lower than those in the Texas colonies. There was also no clear evidence that these observed differences in allelic richness and heterozygosity are associated with a founder event related to species reintroductions. Queen Bess Island was established as a result of the restoration program, whereas the nearby colony on Wine Island was naturally established in 1997 as local populations expanded [82]. In contrast, the population on Raccoon Island was also reintroduced between 1984 and 1986 [42], and yet it shows above-average levels of allelic diversity and heterozygosity relative to other colonies sampled here. Galliard Island’s population, which exhibited relatively low diversity, was naturally founded sometime after the dredge island was created in 1981 [83]. Pelicans were not known to nest in Alabama before approximately the same time [83], and it would not be surprising if the birds that colonized the island came from restored populations of southeastern Louisiana, some of which are half the distance to Gaillard as are the next closest colonies in Florida.

The presence of reduced genetic diversity following reintroduction, along with failure to detect signatures of a bottleneck such as heterozygote excess, are not surprising given that it has been shown that reductions of heterozygosity and allelic richness are more sensitive to founder events than is heterozygosity excess [84]. Low levels of genetic diversity compared to other northern Gulf populations suggest that some of the colonies in Louisiana could have been bottlenecked for several generations. Alternatively, if founders were a non-random subset of the source population, such as multiple cases where chicks from the same nest were released together, heterozygosity might also be reduced. However, it is important to note that none of the measures of genetic variation in Louisiana were significantly lower than the levels observed in the source samples from Florida, so if the low levels of variation we observed at Queen Bess, Wine and Galliard are due to a bottleneck, the reductions were not large. There was also no evidence that birds from these islands are suffering from depressed reproduction that could not be explained by environmental variation [85]. This conclusion is supported by an ecological assessment of the Louisiana population conducted not long after reintroductions ended, in which colonies established via translocation were found to have reproductive rates equal to the regional average [44].

Spatial variation among the populations prior to 2010 suggests a weak gradient in variation in the Gulf, with populations in Texas being differentiated from those in southeastern Louisiana, Alabama, and Florida. These spatial patterns could be the result of limited gene flow along the Gulf coast, but isolation by distance does not appear to drive differences between the Texas populations and those located further east. An alternate explanation is that the population structure prior to 2010 was shaped, in part, due to gene flow between the populations in southeast Louisiana and Florida resulting from the reintroduction event [41,42]. We consider it likely that this structure was reduced by natural dispersal between the Texas sites and those in Louisiana, as is suggested by the intermediate assignment probabilities of Rabbit and Raccoon Islands.

While overall patterns of within-colony genetic diversity are encouraging for the species’ status in the region, the temporal changes in population structure we observed between our two sampling periods are of potential concern. More specifically, we may have detected an apparent decrease in pairwise differentiation and clear structuring across the region immediately following the Deepwater Horizon oil spill, and have at minimum observed a shift in allele frequencies across multiple colonies over essentially one generation [40]. As we are unable to directly test the mechanism of these changes, we present several scenarios, involving both natural and anthropogenic effects, that may explain the observed patterns.

Some effects on genetic diversity and structure may result from basic attributes of brown pelican life history. Based on band recovery efforts, many seabirds are considered to be highly philopatric, returning to natal breeding colonies despite long-distance movements during the non-breeding season [78,86–91]. Brown pelicans have been considered philopatric because banded individuals were resighted predominately on their natal island, but resighting rates were low [92]. In contrast, genetic assessment of seabird populations frequently yields surprisingly low structure [80,93,94]. If brown pelicans have lower site fidelity than has been suggested from banding data, then strong differentiation across the northern Gulf may never have been present. However, we detected some structure, with higher genetic diversity in the Texas populations than those in southeastern Louisiana, and genetic differentiation between the Texas sites and others in Louisiana, Alabama, and Florida. Pairwise differentiation was highly significant between these colonies and several others, and well within both the range at which structure can be discerned using microsatellites [95] and that of other seabirds in which structure has recently been reported [96–98]. It is not clear if this pattern was due to strong site fidelity, or is a remnant of the reintroduction event. In any case, our pre-2010 results do not support the hypothesis that extensive dispersal has resulted in a single panmictic population.

Regarding the Deepwater Horizon oil spill, we predicted that populations in the areas receiving the highest levels of oil would experience decreased genetic diversity, consistent with loss of individuals via abandonment and/or mortality at these sites. The sizes of many of these colonies (hundreds to thousands of nesting pairs in most cases) do not suggest that the predicted levels of variation would occur naturally between sampling events, and would support our initial assumption that the sampling bouts represent two distinct periods of interest. Also, if the oil spill caused widespread abandonment of nest sites in heavily impacted areas from southeastern Louisiana to western Florida, post-2010 genetic structure would be reduced relative to pre-2010 genetic structure. Alternatively, if prior gene flow was sufficient to prevent genetic differentiation among colonies, no observable effects of the spill would be expected.

As predicted, we found considerable changes between the two sampling periods. The geographic component of observed differences in genetic diversity prior to the oil spill was absent in the samples collected post-2010, and temporal variation in population allele frequencies was non-zero in most sites. Furthermore, we found no effect of time between the pre- and post-2010 sampling on change in allele frequencies, despite variable time intervals between sampling periods for these colonies, supporting our assumption that the 2010 spill represents a legitimate point of demarcation between two ecologically relevant time periods. Several processes could explain these observed differences between the two samples. A study of Louisiana colonies over forty years suggested that when conditions at a colony site deteriorate, birds may move en masse to another site [82]. Several Louisiana colonies are known to have experienced severe physical degradation as a result of the spill [30,99] and persistent exposure to oil and oil-based compounds in subsequent years [100]. The degradation of high-quality nesting sites could have played a large role in dispersal of individuals from the area, resulting in increased gene flow between some, but not all pairs of populations. These admixture events might have been localized, with groups of birds colonizing sites that were not saturated with individuals that had been born at the sites. Additionally, over 1,200 oiled brown pelicans were rehabilitated and released at a variety of sites, including several in Texas, Georgia, and Florida, and experienced high survival [101] and some reproduction in 2011 [40]. Along with larger groups of birds abandoning oiled regions, these processes could have made considerable contributions to novel genotype introduction and decreased the geographic patterns in diversity and differentiation that were present prior to the spill.

Another potential driver of our observed patterns is the dynamic nature of the Gulf coast network of breeding colonies, independent of large, punctuated disturbances such as the Deepwater Horizon spill. Coastal land loss and tropical storms have impacted barrier islands to a considerable degree throughout the region, with associated fluctuations in the size of breeding pelican populations [82,102], as well as other species [102–104]. Wine Island faced significant erosion in the late 2000s, and further degradation and disturbance due to oiling and subsequent cleanup efforts [92]. Island loss also occurred throughout the Barataria Bay and Chandeleur Island regions, with many small colonies lost entirely [82]. Without reliable breeding sites for a considerable portion of the regional population, it may be likely that higher rates of gene flow would occur than would be expected given a more stable landscape. These colony losses also occurred in parallel with the growth of the breeding population of Rabbit Island in southwestern Louisiana [82], suggesting that at least some westward movement of breeding individuals may be occurring, perhaps in response to land loss elsewhere. However, knowledge of these processes would predict the presence of large, undifferentiated groups within regions at any point in the decade under observation, and does not account for the breakdown of structuring observed between our two sampling periods. At present, we are unable to entirely decouple the specific influences of oiling, associated human activities, and landscape-level colony impacts in shaping the current patterns we see in the regional brown pelican population, though we consider it likely that each of these drivers contributed to the patterns we have documented.

Our study represents one of the first examples of large change in gene frequencies and population structure in an avian population over a relatively short time [12], thus highlighting the conservation value of relating genetic assessments to management histories in order to assess populations in an integrative fashion. When viewed through one lens, the observed exchange of genetic material at the regional scale is encouraging, as it suggests that local disturbances are unlikely to eliminate unique genotypes in the Gulf through localized extinctions. However, these findings also raise the possibility that extensive movement of individuals could have demographic consequences as more individuals inhabit less-impacted sites and incur potential reductions in fitness due to various density-dependent processes [105]. Additionally, genetic changes in unmonitored seabirds and other species, many of which exhibit smaller generation times than pelicans, could have occurred over the same period, with their own attendant conservation issues. This highlights the importance of establishing baselines in areas where large disturbances are likely to occur to permit accurate impact assessments and restoration goals reflecting pre-disturbance conditions. For example, in a region such as the Gulf of Mexico, which experiences multiple disturbances that act on various spatial and temporal scales, an existing genetic monitoring program that predated our sample collection could have allowed for additional temporal comparisons that would have allowed us to more confidently ascribe our results to an individual event such as the Deepwater Horizon spill. As genetic assessment of populations increase in their genomic coverage and affordability [7,106], more complete genetic profiles may be maintained to gauge effects on the diversity of large regional populations at fine temporal scales.

## Supporting information

S1 File Additional information on sample extraction and microsatellite allele calls. (DOCX)

S2 File Example pre-2010 assignment plots demonstrating a lack of clear support for higher suggested optimal values of K in determining regional population structure. (TIF)
