# Supplementary material for: Genetic structure of brown pelicans (Pelecanus occidentalis) in the northern Gulf of Mexico in the context of human management and disturbance
Source: PLoS One. 2017 Oct 4;12(10):e0185309. doi: 10.1371/journal.pone.0185309 (PMC5627915; doi:10.1371/journal.pone.0185309)
Supplement: S1 File — (DOCX) [file pone.0185309.s001.docx]

**S1 Text. Additional information on sample extraction and microsatellite allele calls.** We created two polymerase chain reaction (PCR) multiplexes using the Qiagen Multiplex PCR Kit: locus Po26, and Po29 (Multiplex A); locus PEL 086, Po14, Po16, and Po17 (Multiplex B). We ran Locus PeEr04 in its own PCR. We amplified multiplexes A and B in a 10μl reaction volume using the Qiagen Multiplex PCR Kit with 5μl 2x Multiplex PCR Mastermix, 1μl 2μM Primer Mix, 2μl dH_2_O, and 2μl DNA. We performed PCRs on an Eppendorf ProS thermocycler using cycling conditions outlined in the PCR Kit, with an annealing temperature of 55°C. We amplified locus PeEr04 in a 15μl reaction volume using 1.5μl 10x Buffer, 1.5μl MgCl_2_, 0.3μl dNTPs, 0.5μl each of forward and reverse primer, 0.08μl Taq Gold, 7.62μl dH_2_O, and 3.0μl DNA. We performed PCRs in the same thermocycler under the following conditions: 95°C for 10 min followed by 35 cycles of 95°C for 30 s, 50°C for 30 s, and 72°C for 45 s, followed by an extension of 72°C for 10 min, and a cooling phase of 25°C for 1 min. We then pooled the two PCR multiplexes and locus PeEr04 and run on an ABI 3100 capillary sequencer. We scored product sizes relative to an internal size standard (LIZ500, Applied Biosystems) using automated allele calling procedures in Genemapper, version 4.0 (Applied Biosystems) software.
