# Supplementary figures and images for: Genetic structure of brown pelicans (Pelecanus occidentalis) in the northern Gulf of Mexico in the context of human management and disturbance
Source: PLoS One. 2017 Oct 4;12(10):e0185309. doi: 10.1371/journal.pone.0185309 (PMC5627915; doi:10.1371/journal.pone.0185309)

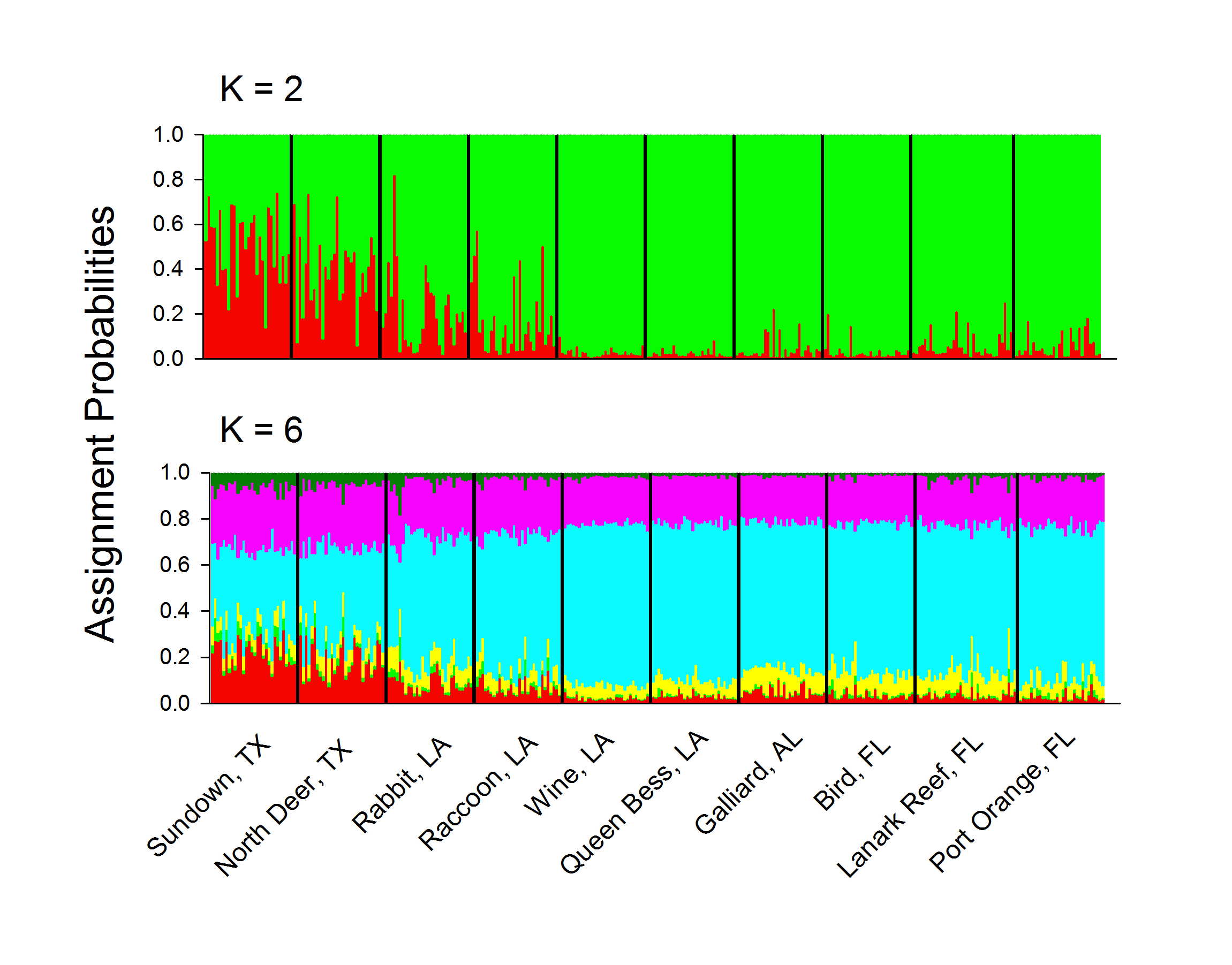

Supplement: S2 File — (TIF) [file pone.0185309.s002.tif]
